# Stringent requirement for spatial arrangement of extracellular matrix in supporting cell morphogenesis and differentiation

**DOI:** 10.1186/1471-2121-15-10

**Published:** 2014-03-25

**Authors:** Sze Wing Tang, Wing Yin Tong, Wei Shen, Kelvin W K Yeung, Yun Wah Lam

**Affiliations:** 1 Department of Biology & Chemistry, City University of Hong Kong, Hong Kong, China; 2 Department of Orthopaedics & Traumatology, The University of Hong Kong, Hong Kong, China; 3 Shenzhen Key Laboratory for Innovative Technology in Orthopaedic Trauma, The University of Hong Kong Shenzhen Hospital, 1 Haiyuan 1st Road, Futian Distract, Shenzhen, China

**Keywords:** Achilles tendon, Mesenchymal stem cells, Extracellular matrix, Collagen, Microenvironment

## Abstract

**Background:**

In vitro experiments on the functional roles of extracellular matrix (ECM) components usually involve the culture of cells on surfaces coated with purified ECM components. These experiments can seldom recuperate the spatial arrangement of ECM found in vivo. In this study, we have overcome this obstacle by using histological sections of bovine Achilles tendon as cell culture substrates.

**Results:**

We found that tendon sections can be viewed as a pre-formed block of ECM in which the collagen fibrils exhibited a spatial regularity unraveled in any artificially constructed scaffold. By carving the tendon at different angles relative to its main axis, we created different surfaces with distinct spatial arrangements of collagen fibrils. To assess the cellular responses to these surfaces, human mesenchymal stem cells (MSCs) were directly cultured on these sections, hence exposed to the collagen with different spatial orientations. Cells seeded on longitudinal tendon sections adopted a highly elongated and aligned morphology, and expressed an increased level of tenomodulin, suggesting that the collagen fibrils present in this section provide a microenvironment that facilitates cell morphogenesis and differentiation. However, MSC elongation, alignment and induction of tenomodulin diminished dramatically even as the sectioned angle changed slightly.

**Conclusion:**

Our results suggest that cell functions are influenced not only by the type or concentration of ECM components, but also by the precise spatial arrangements of these molecules. The method developed in this study offers a simple and robust way for the studying of cell-ECM interactions, and opens many research avenues in the field of matrix biology.

## Background

Extracellular matrix (ECM) is a complex mixture of molecules that exist at the interface between cells in biological tissues. Many ECM proteins consist of conserved functional domains and are heavily decorated with glycosaminoglycans [1,2]. These domains act as the binding sites for other ECM proteins, cellular receptors and soluble growth factors [2]. Over the past decades, knowledge on the functional roles of ECMs have been generated from in vitro experiments, in which cells are co-cultured with purified ECM components in two- or three-dimensions. For example, Schedin et al. [3] used ECMs isolated from rat mammary glands to generate 3D culture scaffolds. Mammary epithelial cells seeded in ECMs isolated from nulliparous rats developed epithelial ducts in vitro, whereas the same type of cells died in ECMs isolated from mid-involuting mammary glands. More recently, ECMs have been found to induce the direct transdifferentiation of bone vascular cells into lymphatic endothelium [4]. Mouse embryonic stem cells (ESCs) seeded onto ECMs isolated from the osteogenic cell line MC3T3E1 differentiate into osteoblasts more efficiently than on ECMs from a non-osteogenic cell line [5]. Similarly, specific combinations of ECM proteins have been shown to direct the differentiation of primary human neural precursor cells into either neurons or glial cells [6]. These remarkable observations suggest that ECMs contain biological cues that can instruct cell fate determination, and different combinations of ECM components may harbor specific signals for particular cell types and for effecting different phenotypic consequences. Furthermore, the density of ECMs is known to influence cell spreading and migration speed [7], possibly due to the effect of ECM concentration on the clustering of integrins on cell membranes [8].

While these in vitro models are powerful in delineating the functions and dosage effect of specific ECM components, much less is known about the biological roles of the spatial arrangement and orientation of individual ECM molecules. Many organs contain ECM components arranged in highly distinctive spatial organizations [9]. Among them, tendons are one of the most structurally organized tissues and the best illustration of the level of structural organization of ECMs in vivo [10]. Tendons are mainly composed of Collagen I, with small amounts of Collagens III and V [11]. The collagen fibres in tendons are bundled as highly organized parallel arrays [12], which serve to transmit force generated from muscle to bone [13]. The building blocks of collagen fibers are triple helix collagen monomers assembled from three collagen polypeptides. Each monomer is composed of two α1 and one α2 collagen chains. The triple-helical monomer is approximately 300 nm in length and 1.5 nm in diameter. Recent studies have indicated that the interaction sites for collagen binding proteins are highly ordered on a three-dimensional structure of collagen fibrils. These ligand-binding sites are assembled on collagen monomers, and depending on how the collagen aggregates, these sites are exposed or hidden [14]. Hence, cell interaction and ligand interaction domains are arranged in a specific pattern on collagen fibres.

A number of techniques have been recently developed to control the spatial arrangement of collagen proteins in artificial scaffolds. Collagen fibres can be physically aligned by electrospinning [15], microfluidics [16,17], molecular crowding [18], electrochemical fabrication [19], magnetic fields [20], strain pulling [21] and extrusion methods [22]. However, the resulting fibrils, albeit aligned to various extents, are still very disorganized when compared to natural collagen fibrils, and lack the architectural complexity observed in naturally occurring ECMs. Nevertheless, the structural alignment of collagen fibres generated by these techniques can affect cell morphology. For example, human fibroblasts seeded on collagen conduits are significantly aligned along the collagen fibril axis [22] and a similar alignment has been observed for bovine aortic endothelial cells cultured on aligned collagen scaffolds produced by microfluidics [16]. Conversely, mesenchymal stem cells (MSC) and rotator cuff fibroblasts cultured on anisotropic substrates secreted aligned collagen fibres [23,24]. These studies suggest the mutual relationship between the physical packing of ECMs and cell morphology, but systematic and quantitative understanding of how the spatial organization of ECMs affects cellular functions is still lacking.

Our group has recently developed a novel approach for studying the functional roles of the tissue microenvironment [25], in which histological sections from intact mammalian tissues are directly used as cell culture substrates. Cells seeded on these sections are exposed to native ECM scaffolds, and adopted morphological and biochemical changes as a result. We have envisaged a mammalian tendon as a relatively acellular block of collagen with a strikingly ordered organization, and can be viewed as a convenient, naturally occurring collagen scaffold for cell culturing. In this study, we have explored this property and created cell culture substrates from bovine Achilles tendon sectioned at different angles. These surfaces contain the same biochemical composition, but different in the arrangement of collagen fibrils. Human MSC were seeded onto these sections, hence exposed to collagen fibrils with different arrangements. This experimental design provides a simple but novel way for the systematic analysis of the influence of ECM arrangements on cell behaviour.

## Results

### Organization of collagen fibrils in self-assembled hydrogels and in natural tendon tissue

**Figure 1:**
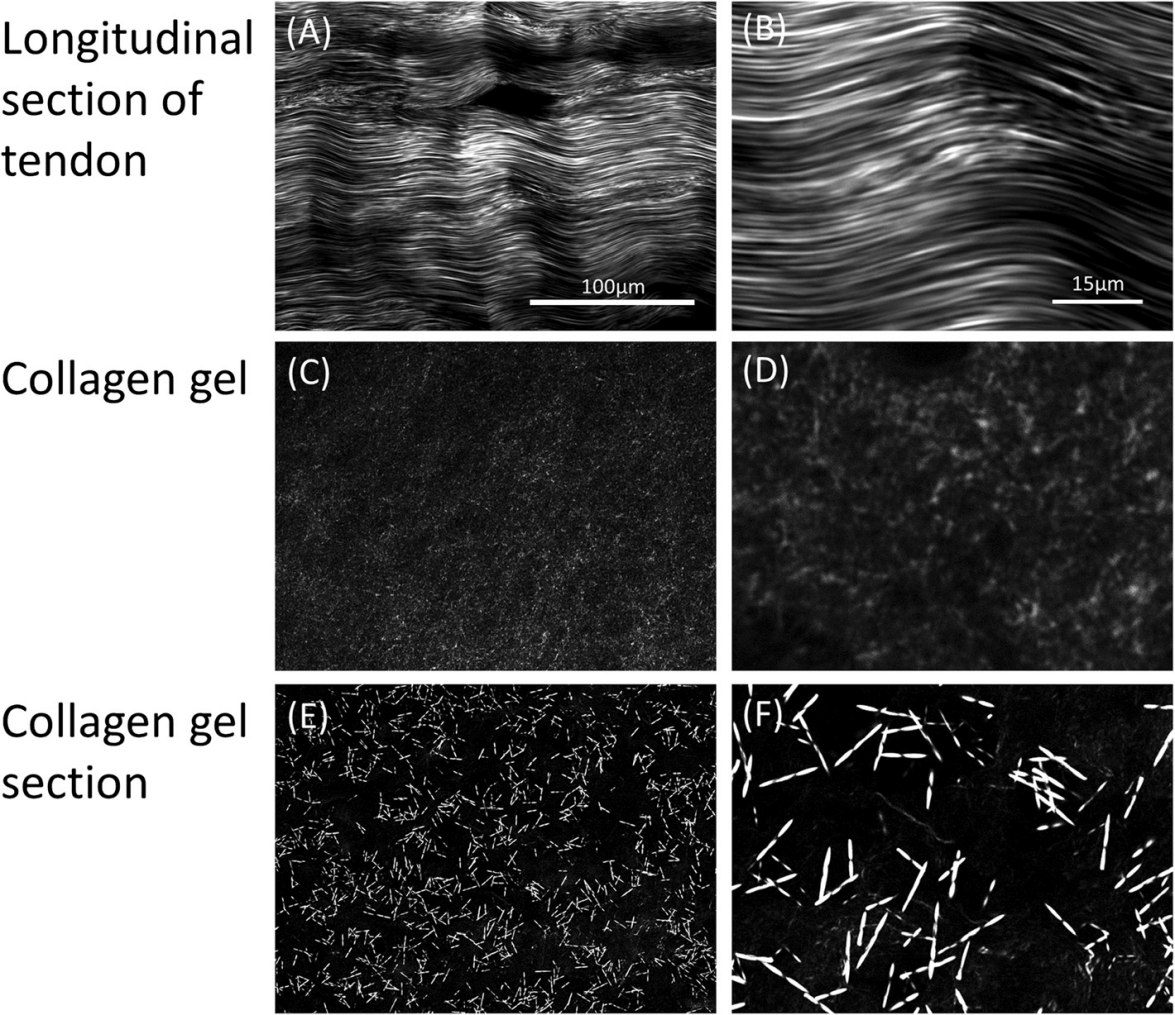
**SHG microscopy images. (A-B)** Longitudinal section of bovine Achilles tendon, **(C-D)** amorphous collagen from direct coating of Collagen I on glass surfaces and **(E-F)** gel Collagen I fibres.

Type 1 collagen is one of the most commonly used ECM components in cell culture experiments in which crudely purified collagen, typically from mammalian tendons, is used to coat cell culture surfaces. In our previous study, we simplified this workflow by directing culturing cells onto a layer of native collagen fibrils on mildly fixed tendon cryosections [25]. Here, we compared the arrangement of collagen fibrils in in-vitro assembled collagen gels and tendon sections by using Second Harmonic Generation (SHG) microscopy. This technique allows the non-invasive assessment of the structural symmetry of macromolecules, and can be used to reveal the hierarchical organization of collagen fibrils [10]. As shown in Figure 1C-1D, Collagen I, which is self-assembled on the glass slide, displays weak and punctate SHG signals, suggesting that the collagen molecules present on the slide mainly consists of amorphous proteins. It is possible that the collagen solution dried up too quickly during the coating procedure, thus leaving insufficient time for the collagen molecules to self-organize into fibrillar structures. To provide more time for fibril formation, we slowly warmed the collagen solution to form a hydrogel, which was then sectioned and examined by SHG microscopy (Figure 1E-1F). Short and thick fibres with strong SHG signals were observed, which indicate that collagen fibrils with high order organization have been formed. However, these fibres were relatively distantly spaced, with no clear alignment with one another. This is very different from the collagen fibrils present in the longitudinally cut tendon section (Figure 1A-1B), in which long, aligned collagen fibrils were closely packed. Hence, the molecular arrangement and orientation of collagen fibrils in the tendon are highly distinct from those in self-assembled collagen matrices commonly used in cell culture experiments.

### Differential display of collagen fibrils

**Figure 2:**
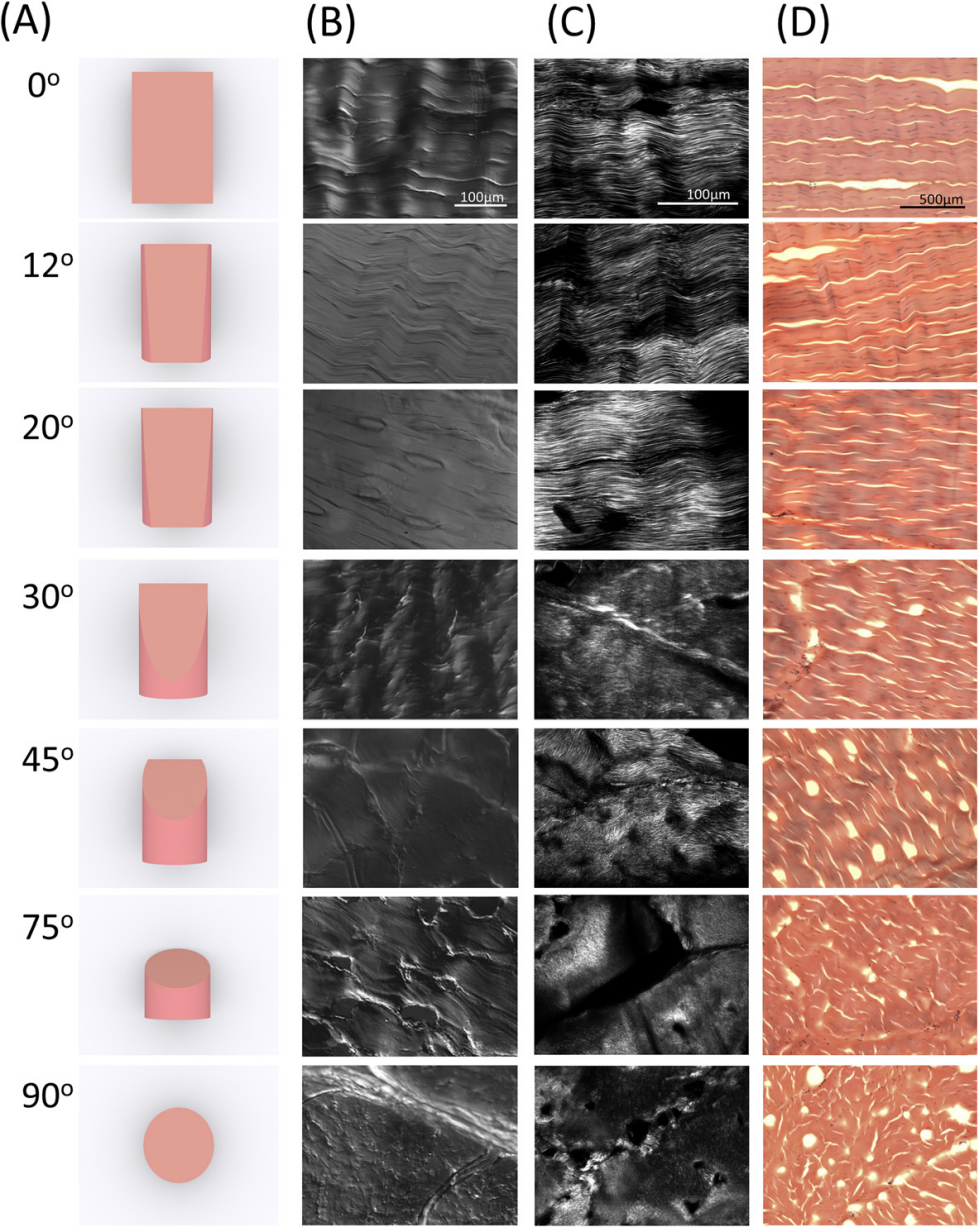
**Histology of cut angle of bovine Achilles tendon sections. (A)** Diagrammatic presentation of cut angle of sections, **(B)** DIC microscopy images, **(C)** SHG microscopy images, and **(D)** hematoxylin and eosin images of bovine Achilles tendon sections with different cut angles.

Achilles tendon is a cylindrical tissue in which collagen fibrils are bundled as precisely aligned fibers arranged along the major axis [26]. By sectioning a block of tendon longitudinally and transversely, we created sections with two distinct histological features [25]. In this study, we have explored this approach by producing seven different types of sections from a single block of tendon. We named these sections according to the cut angles in relation to the major axis. Hence, the longitudinal section (cut along the major axis) is called the “0° section”, and the cross section is called the “90° section”. Between these two angles, we also prepared 12°, 20°, 30°, 45°, and 75° sections (Figure 2A). Differential interference contrast (DIC) microscopy and conventional hematoxylin and eosin staining revealed a gradual transition of the histological characteristics from the longitudinal and transverse cross sections (Figure 2B and 2D). The 0° section had a morphology with a grooved surface, which is consistent with the longitudinal view of the collagen fibril bundles. Each bundle was about 50 μm in diameter, similar to the reported value by [19]. The 90° section, however, appeared to be flatter, possibly representing the horizontal cut surface of the collagen fibrils. The other angles generated surfaces that were in between these two extremes. For example, the 12°, 20°, and 30° sections resembled the 0° section, which is consistent with the oblique cross sections of long cylindrical structures. The SHG microscopy showed consistent results with those of the DIC imagery. Long aligned fibrils were observed in the 0° sections (Figure 2B and 2C), with the 12°, 20° and 30° sections highly similar to the 0° section. As the angle of cutting increased, the length of the fibrils observed in the SHG images decreased. Interestingly, the SHG signals significantly decreased and appeared more disorganized when the cut angle changed from 30° to 45°, thus suggesting that a minor change in collagen fibril length between these two angles results in a significant drop in SHG efficiency.

### Spatial arrangement of collagen fibrils in tendon sections

**Figure 3:**
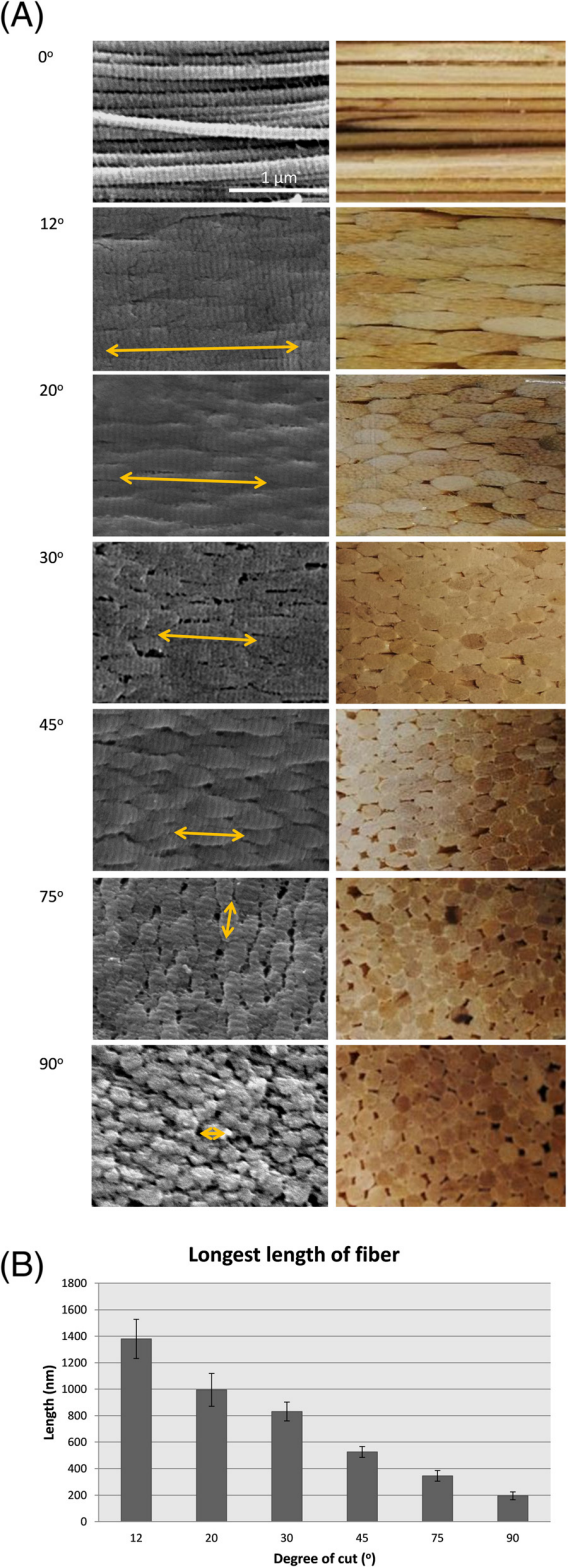
**Quantification of surface of segment of fiber.** (**A**, left panels) SEM images of tendon sections cut at different angles and modeling of tendon sections. Yellow arrows indicate size of a segment of fibers. The length of segment of fibers decreases as cutting angle increases. (**A**, right panels) Model of the various cut sections based on tightly packaged bundles of wooden sticks of uniform size. **(B)** Quantification of surface of longest length of segment of fibers; note significant difference between angles. *p < 0.05.

To further investigate the organization of the collagen fibrils in these sections, we examined their surface topography by using SEM (Figure 3A, left panels). On the 0° section, distinct parallel collagen fibers with a classical banding pattern were observed. The 90° section contained a regular array of round structures, likely representing the cross sections of individual collagen fibers. The other cut angles showed an interesting transition between these two extremes, with the segments of fibers increasingly shorter as the cut angle increased. The observed morphology of these sections is consistent with the view of the tendon as an array of tightly packaged, parallel fibres of homogenous dimension, as illustrated by a bundle of wooden sticks (Figure 3A, right panels). The average lengths of the segment are inversely correlated to the cut angle (Figure 3B), thus indicating that these segments likely correspond to the oblique cross sections of the collagen fibrils. Furthermore, these cross sections in the 12-75° sections appeared to have a staggered arrangement (Figure 3A), which is consistent with the high packing efficiency of the collagen fibrils, as illustrated by the cross sections of a bundle of closely packed wooden rods (Figure 3A). Taken together, this simple and convenient approach of differential cutting of tendon produces surfaces with distinctive arrangements of ECM components. Since these surfaces were carved from the same tissue, their biochemical composition is the same. Therefore, they provide a unique experimental model for systematically studying the roles of the molecular orientation of ECMs in regulating cellular behaviours.

### Influence of collagen fibril arrangement

**Figure 4:**
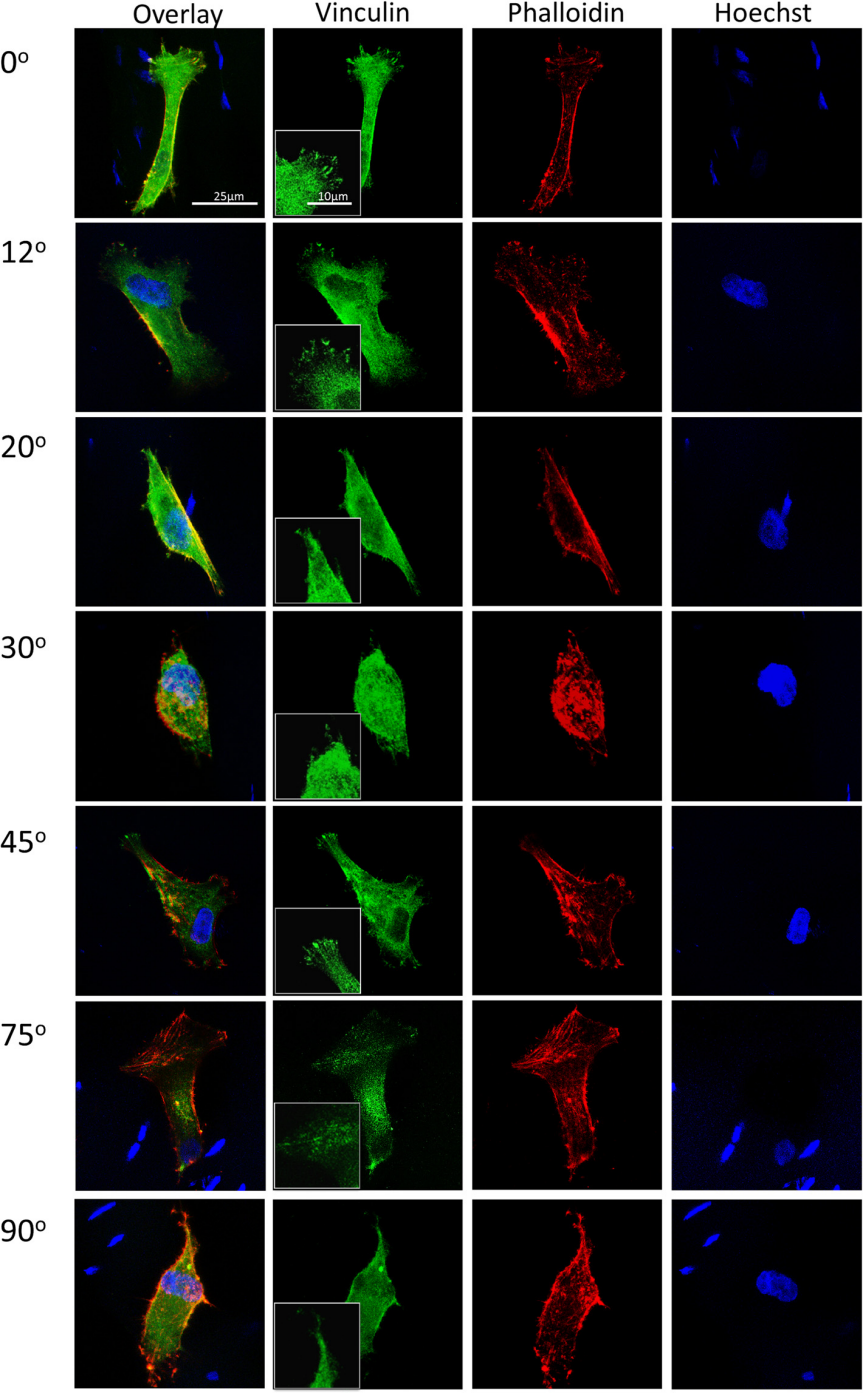
**Focal adhesions of MSCs on tendon sections.** Green signals represent vinculin staining, red signals represent phallodin staining and blue signals represent Hoeschst DNA staining.

**Figure 5:**
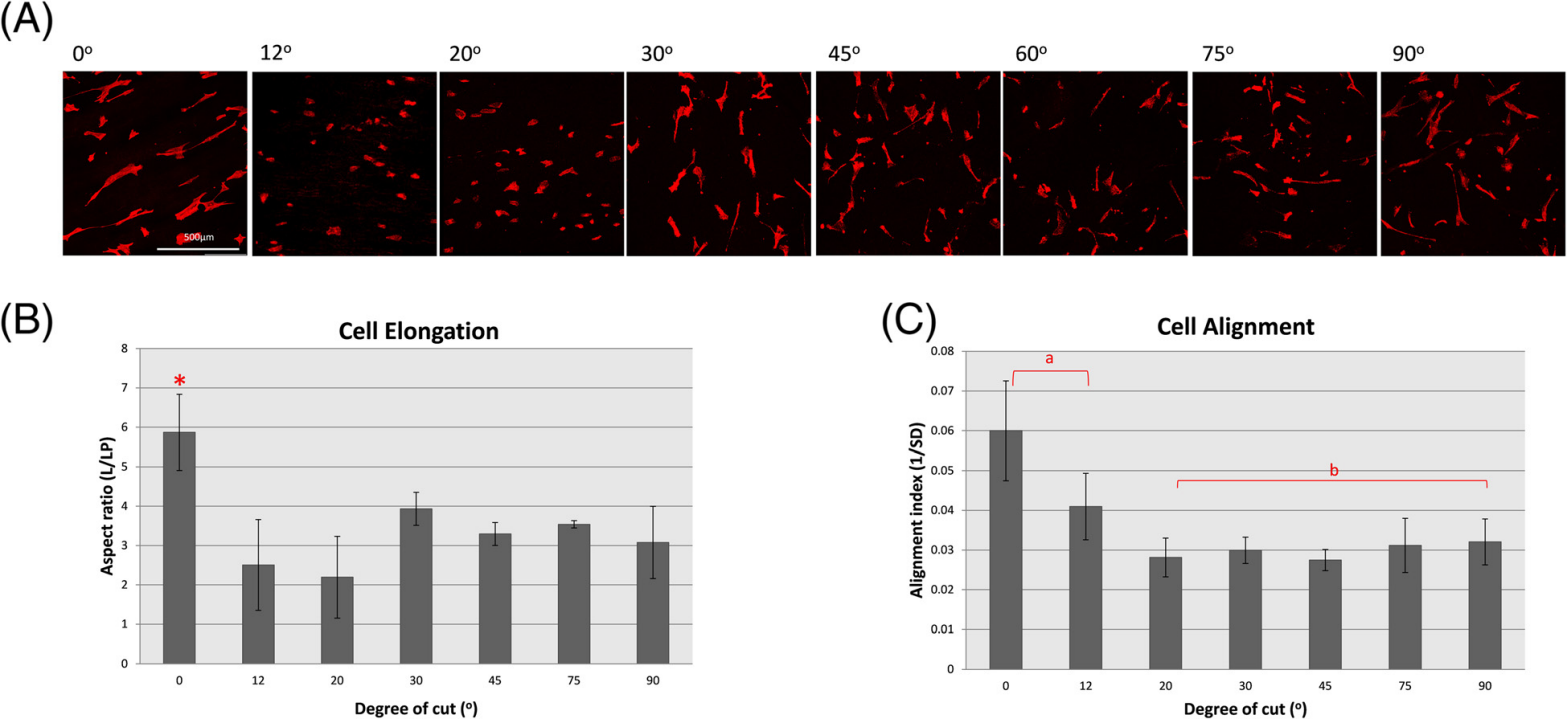
**MSCs elongation and alignment. (A)** Images of MSCs seeded on tendon sections. Red signals represent phallodin staining and blue signals represent Hoechst DNA staining. **(B)** Quantification of cell elongation. **(C)** Quantification of relative cell alignment.

Cells interact with ECM through focal adhesions mediated by transmembrane receptors [27]. We hypothesized that the change in the arrangement of collagen fibrils can be used to alter the accessibility of integrin binding sites and alter the focal adhesion formation. To test this hypothesis, cells were cultured on different sections and stained for vinculin and phalloidin to locate focal adhesion sites. As shown in Figure 4, the cells on the 0° and 12° sections have larger and more defined focal adhesions while those on the 20°, 30°, 75° and 90° sections only have disperse vinculin signals (Figure 4). However, many cells on the 45° section exhibited discrete focal adhesions (Figure 4), suggesting that this cutting angle may have exposed the ECM in such an orientation that favoured focal adhesion formation. The observed discrepancy of vinculin staining pattern did not correlate with the apparent cell morphology. We then assessed the alignment of the MSCs to each another on oblique tendon sections (Figure 5A) and observed that the cells on the 0° and 12° sections are more significantly aligned than on the other cut angles (Figure 5C). This result suggests that the formation of stable focal adhesive complexes may be important for the alignment of MSCs. We also measured the aspect ratios of cells on these sections. Remarkably, the cells on the 0° section were more significantly elongated than those on all of other cut sections (Figure 5B). Although the ultrastructure of the collagen fibrils on the 12° and 20° sections is similar to that on the 0° section (Figure 2B and 2C), the cells adopted a drastically different morphology once the cut angle deviated from 0°. Hence, there appears to be a stringent requirement of collagen fibril arrangement for the elongation and alignment of MSCs. Interestingly, despite the more pronounced vinculin foci detected in cells cultured on the 45° section, we failed to observe any statistically significant difference in cell morphology of these cells from cells on 20°, 30°, 75° and 90° sections.

### Effect of collagen arrangement on tenomodulin expression of mesenchymal stem cells

**Figure 6:**
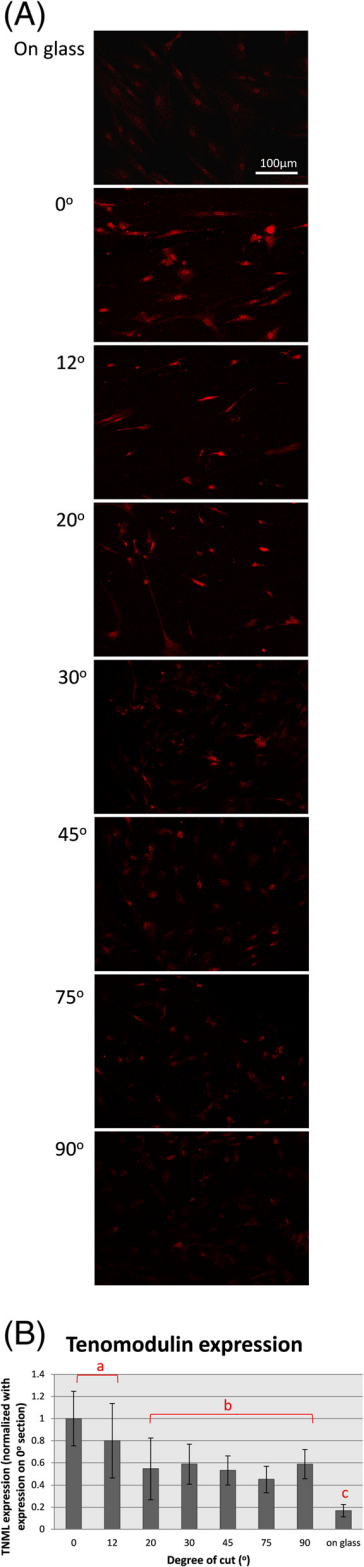
**MSCs tenocytic differentiation. (A)** Tenomodulin staining of MSCs seeded on tendon sections after 12 days of incubation. **(B)** Quantification of staining intensity of tenomodulin in MSCs on tendon sections.

MSCs cultured on longitudinal tendon sections have been shown to express tenomodulin, a well-known tenocyte-specific protein [25]. Here, we compared the levels of tenomodulin expression in MSCs cultured on tendon sections cut at different angles (Figure 6A). The anti-tenomodulin staining intensity of cells grown on a glass surface was generally lower than those on the tendon sections. Among the sections, the 0° and 12° sections appeared to induce a higher level of tenomodulin compared to the other angles (Figure 6B). The result suggests that an incorrect topography fails to promote tenocyte differentiation in MSCs.

## Discussion

This study demonstrates the use of a novel experimental model in the study of the spatial arrangement of ECM components in a cell culture. MSCs seeded on 0° sections adopted a highly elongated and aligned morphology, and expressed a tenocytic marker. The 12° section promoted a similar cell alignment and tenocyte differentiation, but the cells seeded on sections with any other type of angle failed to show these types of behaviors. It is a remarkable observation, as a relatively small (20°) deviation in the sectioned angle can already completely prevent morphological and functional changes in MSCs demonstrated on the longitudinal section. Since the sections are from the same tendon, the biochemical information is identical. Therefore, our results suggest that it is not only the type or concentration of ECM components, but also the precise spatial arrangements and orientation of these molecules are crucial for regulating cell functions. In developing tissue engineering scaffolds, it is important not only to use biomaterials with the suitable biochemical, biocompatibility and biodegradability properties, but also to fabricate them in the correct spatial format [28].

Numerous studies have established the functional roles of ECM components and their interactions with cells and other matrix components. However, the roles of the spatial organization of ECM molecules on cell physiology are often overlooked. We have previously shown that MSCs responded to longitudinal tendon sections by adopting an aligned and elongated morphology [25]. Previous studies have demonstrated that cell alignment is determined by the geometric cues of the aligned fibers [29]. However, our result suggests that MSC alignment may require more than a substrate with aligned topography. The 0°, 12°, and 20° sections all have aligned fibers (Figure 2B and 2C), albeit presented as shorter segments (Figure 3A and 3B). Interestingly, the alignment and elongation of MSCs rapidly diminished when the sectioned angle deviated from 12°, even though the surface topography of the 12° and 20° sections was not dramatically different. We also noted that the MSCs exhibited more well-defined focal adhesions on the sections created by some cutting angles, such as 0°, 12° and 45°, whereas cells on the 20°, 30°, 75° and 90° sections only showed dispersed vinculin signals. Hence, the formation of focal adhesions appeared to be influenced by the geometric features of collagen fibrils. It is possible that MSCs need continuous collagen fibrils of suitable length in order to commit to elongation and differentiation, as suggested by [30]. In the 0° section, the collagen fibrils had comparatively infinite lengths while in the other sections, only relatively short stretches of fibrils were present. A recent study has mapped out all known ligand binding sites on Collagen I fibrils [31], and suggests that on a collagen fibril, cell interaction and matrix interaction domains are alternately placed at regular intervals. It is probable that the placing of these domains plays an important role in cell morphology and differentiation.

Consistent with other previous studies [32-34], our data indicated that the conditions that lead to MSC elongation also lead to tenocytic differentiation. However, the causative correlation between cell elongation and tenomodulin expression is unclear. It is possible that cell elongation is a necessary pre-requisite for tenocytic differentiation, but previous studies by us and other groups have indicated that the sole induction of cell elongation is insufficient to ensure tenocyte differentiation [25,35]. MSCs cultured on silk scaffold with mechanical stress stimulation express a higher level of tenocytic differentiation markers than MSCs cultured on only silk scaffold [36]; the overexpression of scleraxis followed by mechanical stress have a synergistic effect in tenocyte differentiation [37]; Adipose-derived stem cells seeded on aligned collagen fibril with platelet-derived growth factors (PDFGs) has a higher expression of tendon markers than those seeded on aligned collagen fibril alone [38]; and our group previously showed that the correct substrate topography plus the right ECMs were needed to induce MSCs to tenocytic differentiation [25]. These studies suggest that morphological transformation, through the exposure of cells to a permissive microenvironment, may represent an essential step towards tenocytic differentiation in vitro, but other inducing factors are also necessary.

## Conclusion

In this study, we have demonstrated the important role that the precise molecular orientation of collagen plays in regulating cell morphology and differentiation. This implication had been previously neglected, as it has been technically difficult to control the precise location and orientation of ECM proteins when coating cell culture substrates. ‘Pre-formed’ biological scaffolds, as demonstrated from the use of tendon sections here, as ECM coating can circumvent this problem. The method developed in this research offers a simple and robust way for the studying of cell-ECM interactions, and opens many research avenues in the field of matrix biology.

## Methods

### Cell culture

TERT-immortalised human mesenchymal stem cells (MSCs) expressing GFP stably were a gift from Dr. Dario Campana (St. Jude Children’s Research Hospital, Memphis, USA). The cells were cultured at 37°C in 5% CO_2_ in a humidified incubator. Dulbecco’s modified Eagle’s medium (DMEM) low glucose (Invitrogen, 31600–026) supplemented with 10% heat inactivated fetal calf serum (Caisson, FBL02-500 ML), and 1% Antibiotic-Antimycotic (Invitrogen, 15240) was used. The culture media were refreshed every three days and cell confluence was always kept under 80%. The cells were seeded at 10^4^ cells/cm^2^ onto the tendon sections and maintained in the same culture medium as the culture.

### Tendon sections

Bovine Achilles tendon was bought from a local wet market and stored at −80°C. The tendon was trimmed into tendon blocks of different angles (0°, 12°, 20°, 30°, 45°, 75° and 90°), as shown in Figure 2 before performing cryosectioning. The tendon block was mounted onto a cryostat tissue holder (Jung CM 1500, Leica Instruments) with a tissue freezing medium (OCT, Jung, 0201 08926). Cryosections of 25 μm in thickness were thawed onto glass slides (Menzel-Glaser Superfrost Ultra Plus). Rectangles (5 × 2.5 cm^2^) around the sections were drawn on the slide with a hydrophobic blocker (Mini PAP Pen, Invitrogen) in order to standardize the cell seeding areas. The sections were washed with 1x phosphate buffered saline (PBS) and fixed with 4% paraformaldehyde (PFA) (Sigma-Aldrich, 30525-89-4) in 1x PBS for 15 minutes at room temperature. The slide was than washed with 1x PBS for 10 minutes and followed by glycine quenching (100 mM glycine in PBS, USB) two times (15 minutes each time). The slides were further washed two more times with 1x PBS (10 minutes each time).

### Collagen gel sections

DMEM (50 μl, 10x) and 50 μl 10x reconstitution buffer (262 mM NaHCO_3_ and 77 mM HEPES) were added into 400 μl of 5 mg/ml Collagen I (Invitrogen, A10483-01). The mixture was put into a 1.5 ml centrifuge tube. The centrifuge tube was incubated at 37°C for 30 minutes to form solid collagen gel. The centrifuge tube which contained the solid collagen gel was frozen at −80°C for at least 3 hours before cryosectioning. The collagen gel was taken out of the tube and mounted onto a cryostat tissue holder (Jung CM 1500, Leica Instruments) with a tissue freezing medium (OCT, Jung, 0201 08926). Cryosectioning was performed as described above.

### Cell staining

The cells were seeded on the sections as described and cultured for 3 days. After 3 days, the cells were washed twice with pre-warmed (37°C) 1x PBS and then fixed with 4% PFA for 15 minutes in room temperature. In some of the experiments, the cells were stained with Rhodamine-phalloidin (Invitrogen, R415) and Hoechst (Sigma Aldrich, B2261) as previously described [23]. For immunofluorescence, the cells were fixed in freshly prepared 4% PFA fixation for 15 minutes at room temperature, washed with 20 ml PBS, than quenched with 100 mM glycine in PBS for 15 minutes. After quenching, the cells were washed twice with PBS (5 minutes each time) and permeablized in 1% Triton X-100 in PBS for 2 minutes at room temperature. The cells were washed with PBS twice (5 minutes each time) and blocked with 1% bovine serum albumin (BSA) in PBS for 30 minutes at room temperature. The cells were incubated with a primary antibody for 120 minutes followed by three PBS washings (5 minutes each time) before incubated with a fluorochrome-conjugated secondary antibody for 120 minutes. The primary antibody used was rabbit anti-tenomodulin (Santa Cruz, SC-98875) and vinculin (Millipore, MAB3574), and the secondary antibody used was Alexa 647 donkey anti rabbit (Invitrogen, A31573) and Alexa 488 goat anti mouse (Invitrogen, A11001). After incubation with the antibodies, the cells were washed twice with PBS (5 minutes on rocker) and counter stained with Hoechst for labeling the nucleus. The stained samples were imaged with a laser scanning confocal microscope (Leica TCS SPE).

### Histological staining

The tendon sections were prepared as described. The samples were rehydrated in 1x PBS for 1 hour, then washed with running tap water for 1 minute. The samples were than stained with Gill’s hematoxylin No. 3 (Electron Microscopy Sciences, 26030–30) for 2 minutes, washed with running tap water for 1 minute, than blued with 0.2% ammonia water for 1 minute followed by washing with running tap water for 1 minute. The samples were than stained with eosin (Electron Microscopy Sciences, 14851) for 1 minute followed by a series of dehydration with 95% and 100% ethanol. The samples were cleared with xylene and mounted with Permount (Fisher Scientific, SP15-500).

### Scanning electron microscopy

The tendon sections were prepared as described above. The sections were fixed with 3% glutaraldehyde in 1x PBS for 15 minutes. The fixed samples were dehydrated with a series of ethanol with increasing concentrations (30%, 50%, 70%, 80%, 90%, 95% and 100%) (5 minutes each time). After treatment with ethanol, the sections were further dehydrated with 100% ethanol and 100% acetone in ratios of 3:1, 1:1 and 1:3 (10 minutes each time). Afterwards, the sections were treated with 100% acetone three times (15 minutes each time). The entire dehydration process was completed under room temperature. CO_2_ critical point drying was carried out by using a BAL-TEC CPD 030 critical point dryer at 10°C for 120 minutes. The samples were than mounted onto an aluminum stub with carbon tape and coated with carbon by evaporation. The carbon-coated sections were imaged by using an environmental scanning electron microscope (FEI/Philips XL30 Esem-FEG).

### Second harmonic generation microscopy

The tendon and collagen gel sections were prepared as described. Unfixed sections were immersed with 1x PBS and imaged under a second harmonic generation (SHG) microscope (Leica TCS SP5 laser scanning microscope with Coherent Chameleon Ultra II multi-photon laser and Leica NDD DAPI detector).

### Image analysis

Cell number, cell alignment, elongation and cell differentiation were quantified by using NIH ImageJ (version 1.42q). For cell alignment quantification, a reference line was randomly defined. The major axis of the cells was defined. The angle between the major axis and reference line was measured. The relative alignment was reported. The average aspect ratio of the cells (long axis/short axis) was measured to quantify cell elongation. At least 50 cells were randomly selected from each sample for analysis. For cell differentiation, the intensity of the tenomodulin staining was quantified by using NIH ImageJ. The cells were first recorded as a region of interest (ROI), and the ROI was defined by whole cell staining. The number of ROIs was recorded.

### Statistics

The data were tested with an analysis of variance (ANOVA) to determine if there were any significant differences between each treated group.

## Abbreviations

BSA: Bovine serum albumin; DMEM: Dulbecco’s modified Eagle’s medium; ECM: Extracellular matrix; MSCs: Mesenchymal stem cells; PBS: Phosphate buffered saline; PFA: Paraformaldehyde; SHG: Second harmonic generation.

## Competing interests

The authors declare that they have no competing interests.

## Authors’ contributions

SWT carried out the whole study, including histological studies, cell culture experiments, imaging, data analysis and drafted the manuscript. WYT participated in cell culture experiments, imaging and data analysis. WS participated in imaging and data analysis. WYT and YWL conceived of the study, and participated in planning, design and coordination and help with manuscript preparation and editing. All authors read and approved the final manuscript.
